# Evaluation of Nonpharmacologic Interventions and Sleep Outcomes in Hospitalized Medical and Surgical Patients: A Nonrandomized Controlled Trial

**DOI:** 10.1001/jamanetworkopen.2022.32623

**Published:** 2022-09-21

**Authors:** Eva S. van den Ende, Hanneke Merten, Lisanne Van der Roest, Belle Toussaint, Quirine van Rijn, Marjolein Keesenberg, Anne M. Lodders, Kim van Veldhuizen, Iris E. Vos, Sophie Hoekstra, Prabath W. B. Nanayakkara

**Affiliations:** 1 Section of General Internal Medicine, Department of Internal Medicine, Amsterdam Public Health Research Institute, Amsterdam UMC location Vrije Universiteit Amsterdam, Amsterdam, the Netherlands; 2 Department of Public and Occupational Health, Amsterdam Public Health Research Institute, Amsterdam UMC location Vrije Universiteit Amsterdam, Amsterdam, the Netherlands

## Abstract

**Importance:**

Inadequate sleep negatively affects patients' physical health, mental well-being, and recovery. Nonpharmacologic interventions are recommended as first-choice treatment. However, studies evaluating the interventions are often of poor quality and show equivocal results.

**Objective:**

To assess whether the implementation of nonpharmacologic interventions is associated with improved inpatient night sleep.

**Design, Setting, and Participants:**

In a nonrandomized controlled trial, patients were recruited on the acute medical unit and medical and surgical wards of a Dutch academic hospital. All adults who spent exactly 1 full night in the hospital were recruited between September 1, 2019, and May 31, 2020 (control group), received usual care. Patients recruited between September 1, 2020, and May 31, 2021, served as the intervention group. The intervention group received earplugs, an eye mask, and aromatherapy. Nurses received sleep-hygiene training, and in the acute medical unit, the morning medication and vital sign measurement rounds were postponed from the night shift to the day shift. All interventions were developed in collaboration with patients, nurses, and physicians.

**Main Outcomes and Measures:**

Sleep was measured using actigraphy and the Dutch-Flemish Patient-Reported Outcomes Measurement Information System sleep disturbance item bank. Other outcomes included patient-reported sleep disturbing factors and the use of sleep-enhancing tools.

**Results:**

A total of 374 patients were included (222 control, 152 intervention; median age, 65 [IQR, 52-74] years). Of these, 331 were included in the analysis (195 [59%] men). Most patients (138 [77%] control, 127 [84%] intervention) were in the acute medical unit. The total sleep time was 40 minutes longer in the intervention group (control: median, 6 hours and 5 minutes [IQR, 4 hours and 55 minutes to 7 hours and 4 minutes]; intervention: 6 hours and 45 minutes [IQR, 5 hours and 47 minutes to 7 hours and 39 minutes]; *P* < .001). This was mainly due to a 30-minute delay in final wake time (median clock-time: control, 6:30 am [IQR, 6:00 am to 7:22 am]; intervention, 7:00 am [IQR, 6:30-7:30 am]; *P* < .001). Sleep quality did not differ significantly between groups. For both groups, the main sleep-disturbing factors were noises, pain, toilet visits, and being awakened by hospital staff. Sleep masks (23 of 147 [16%]) and earplugs (17 of 147 [12%]) were used most. Nightly vital sign checks decreased significantly (control: 54%; intervention: 11%; *P* < .001).

**Conclusions and Relevance:**

The findings of this study suggest that sleep of hospitalized patients may be significantly improved with nonpharmacologic interventions. Postponement of morning vital sign checks and medication administration rounds from the night to the day shift may be a useful way to achieve this.

**Trial Registration:**

Netherlands Trial Registry Identifier: NL7995

## Key Points

**Question:**

Are nonpharmacologic interventions, such as postponed measuring of morning vital signs, sleep hygiene training for nurses, and routine distribution of earplugs and sleep masks associated with improved sleep in patients hospitalized in medical and surgical units?

**Findings:**

In this nonrandomized controlled trial including 374 inpatients, a significant difference of 40 minutes in total sleep time was found in favor of the intervention group. This improvement was associated with a 30-minute delay in final wake time.

**Meaning:**

The findings of this study suggest that postponement of early morning nursing rounds may have utility as a sleep-enhancing intervention.

## Teaser

This nonrandomized controlled trial examines the use of nonpharmacologic interventions to improve the length and quality of sleep in hospitalized patients

## Introduction

### Background

Hospitalized patients often have difficulty with sleep.^1^ Inadequate sleep is negatively associated with both physical and mental health,^2,3,4,5^ including respiratory, endocrine, and metabolic function^6,7,8,9,10,11,12^; immunosuppression^13^; delayed wound healing and increased insulin resistance^2,14,15^; risk of fall incidents^16^; delirium^17,18,19^; and death.^20^ Furthermore, inadequate sleep appears to affect mood,^15^ cognitive performance (eg, memory and decision-making),^21,22^ and pain perception.^23^ These issues underscore the importance of research targeting sleep-enhancing interventions in hospitals.

Popular pharmacologic interventions to improve sleep are use of benzodiazepines and melatonin. However, there is insufficient evidence for the success of these medications in hospitalized patients.^24^A systematic review^24^ and meta-analysis^25^ reported that the benefits often do not outweigh the risks of medication.

Because of this insufficient outcome, nonpharmacologic interventions are recommended,^26^ targeting both personal factors (eg, physical and mental discomfort) and environmental factors (eg, noise and light).^1,27,28^ Studies providing patients with earplugs, sleep masks, massages, and education on sleep hygiene reported conflicting results.^29,30,31,32^ Most of these studies are conducted in intensive care units, limited to a specific subpopulation, contain small sample sizes, or focus on a single intervention, ignoring the often multifactorial cause of sleep disturbance.^33,34,35^ In addition, patients are often extensively encouraged to use the offered interventions during the study, so the results may not reflect the outcome in daily practice.^36,37,38^ Therefore, the aim of this study was to evaluate multilevel sleep-enhancing interventions in medical and surgical care wards with voluntary use of offered interventions.

## Methods

### Study Design

This nonrandomized controlled trial, a 19-month, single-center study, was conducted in the acute medical unit, a medical unit (internal medicine/nephrology unit), and a surgical unit (vascular diseases/urology unit) of the Amsterdam UMC location Vrije Universiteit Amsterdam, an academic hospital in the Netherlands.

Given the nature of the interventions, randomization and blinding were not possible and a nonrandomized controlled trial was found to be most suitable. In phase 1 (September 1, 2019, to May 31, 2020), data were collected from patients receiving usual care (control group). Phase 2 (June 1, 2020, to August 31, 2020) was used for the development of sleep-enhancing interventions in collaboration with patients, nurses, and physicians. In phase 3 (September 1, 2020, to May 31, 2021), sleep-enhancing interventions were implemented and continuously carried out while data were collected from newly included patients (intervention group).

The medical ethics review committee of VU University Medical Center approved the study and decided that the Medical Research Involving Human Subjects Act did not apply to this study. The protocol is available in Supplement 1. This report follows the Transparent Reporting of Evaluations With Nonrandomized Designs (TREND) reporting guideline for nonrandomized controlled trials.

### Patients

Patients were eligible for inclusion if they were aged 18 years or older, admitted to 1 of the participating wards, and had spent exactly one night in the hospital (admitted before 3:00 am). Patients were excluded if they were scheduled to stay less than 24 hours on a participating ward, stayed in an infection isolation room, or were unable to speak Dutch or sign written informed consent forms (eg, in case of severe illness or cognitive dysfunction).

### Development of Interventions

Sleep-enhancing interventions were developed and implemented simultaneously on 3 levels: patients, clinicians, and the hospital system. Four patients participated in developing the interventions and information material for patients. This process resulted in a sleep folder with information about the importance of sleep and sleep hygiene, tips and tricks to optimize sleep, and a list of available tools to facilitate sleep. In addition, the folder contained earplugs, a sleep mask, aromatherapy (lavender oil), caffeine-free tea, and quick response codes for mindfulness, audiotape-guided imagery, and relaxing music. In addition, the folder included a description of the theoretical basis for all tools offered. eTable 1 in Supplement 2 provides an overview of the tips and tricks and eFigure 1 in Supplement 2 provides an overview of the sleep folder.

Nurses attended several training sessions on the importance of sleep and sleep hygiene. Knowledge about the physiologic characteristics and importance of sleep were tested with interactive quizzes. In brainstorm sessions, ward-specific interventions were developed and posters were created, which were hung in visible places on the wards (eFigure 2 and eTable 2 in Supplement 2).

Morning medication and vital sign check rounds were changed from the night shift (5:30-7:30 am) to the day shift (7:30-8:45 am). This change in ward logistics was limited to the acute medical unit, where most of the patients included in this study stayed. The medical unit had shifted these activities to the morning rounds long before the start of this study. The surgical unit continued to perform the medication and vital sign check rounds during the night shift. Changes were implemented 2 weeks before the start of the intervention phase. The new schedule was well received by the nurses and physicians and remained after the study had ended.

### Procedures

Seven days per week, for up to 4 times a day during usual working hours, a member of the research team approached all potential eligible patients. After signing informed consent, patients received a sleep registration bracelet (ActiGraph wGT3X-BT [sample rate 30 Hz, epoch 10, and axis 3], ActiGraph LLC),^39^ and sleep folder. The sleep registration bracelet is an electronic noninvasive device that measures sleep by detecting motion with linear accelerometers in 3 axes.^40^ The sleep registration bracelet was worn on the nondominant wrist; if that side was cluttered by intravenous lines, the bracelet was worn on the dominant wrist. Study metrics began after the first hospital night and continued for up to 5 successive nights. Discharge or transfer to a nonparticipating ward were reasons for early dropout. Questionnaires concerning the previous night’s sleep were completed before 11:00 am to minimize recall bias. Seven days per week, a member of the research team visited all participating patients to check whether assistance was required.

All data were collected in an electronic database (maintained by Castor EDC), complying with the European Data Protection Directive and ICH-GCP.^41^ An audit trail was automatically created. All data were coded with record numbers generated by Castor EDC. Only the research team had access to the identifying data.

### Measurement and Instruments

#### Sleep Quantity

Actigraphy provided information about the sleep-onset latency, efficiency, total sleep time (TST), wake after sleep onset, and number and median duration of awakenings. Data were analyzed using ActiLife 6 software, version 6.13.3 (ActiGraph LLC). Cole-Kripke algorithms were applied to optimize prediction of sleep-wake scores.^42,43^ Questions from the Consensus Sleep Diary provided information about sleep and wake time, and subjective number of awakenings.^44^

#### Sleep Quality

The Dutch-Flemish Patient-Reported Outcomes Measurement Information System (PROMIS)–Sleep Disturbance 8b (Short Form, version 1.0) was used to assess sleep quality.^45^ This form has a greater measurement precision and fewer items than the often-used Pittsburgh Sleep Quality Index and Epworth Sleepiness Scale, and has been validated in the Dutch language and population.^46,47^ The questionnaire was minimally adjusted with the phrase *in the past 7 days* being changed to *last night* for all questions. All 8 items are rated on a 5-point Likert scale. The total sum of all scores ranges between 8 and 40, with higher scores indicating more sleep disturbance. Total scores can be converted to standardized T scores, with a T score of 50 being the mean in the reference population.^48^

#### Sleep-Disturbing and -Enhancing Factors

Factors causing sleep onset latency, nocturnal awakenings and final awakenings and the use of sleep-enhancing interventions were recorded using multiple choice and open-text questions.

#### Baseline Characteristics and Secondary Outcome Measures

Baseline characteristics included variables such as age, sex, number of patients in the room, and prehospital insomnia. Secondary outcome measures included the use of sleep medication, length of stay, discharge diagnosis, and hospital readmission collected from the medical record 30 days after inclusion.

### Sample Size

Sample size was calculated based on the results of a large observational study in 39 Dutch hospitals, reporting a subjective mean (SD) TST of 364 (148) minutes.^1^ To detect a clinically relevant 2-sided difference in TST of 10% (36.4 minutes), with an 80% power, α < .05, and 5% dropout rate after inclusion, a sample size of 286 patients in each group was required. A post hoc power analysis revealed a power of 87% after the study was completed.

### Statistical Analysis

Sleep quality and quantity were compared between the control and intervention groups. Confounding and effect modification were assessed using multiple linear regression analysis. Normality was checked by visual inspection of histograms and Q-Q plots. A *P* value of <.05 was considered statistically significant. Analyses were conducted using SPSS for Windows, version 28 (SPSS Inc).

Analysis was primarily performed on the data collected on the first night of participation, because of the considerable decrease in the sample size during each successive day (Figure) and after Friedman tests were conducted to exclude differences in sleep across consecutive nights. Owing to the arrival of COVID-19 in March 2020 in the Netherlands and the associated changes in some of the hospital departments, only the 3 main wards continued participation. To align the control and intervention groups regarding the participating wards, 43 control patients recruited on other wards (ie, plastic surgery, gastroenterology, oncology) were excluded from the main analysis. Results including these patients can be found in eTable 3 in Supplement 2.

**Figure.**
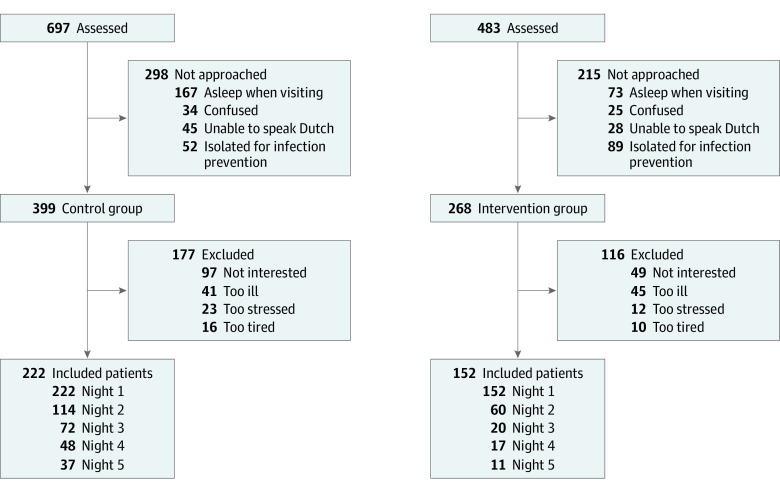
Study Flow Diagram

## Results

A total of 374 patients were included (control, 222 [59%]; intervention, 152 [41%]) (Figure). On the 3 wards that participated in all phases of the study (selected wards), 331 patients were included, with 179 in the control group (54%) and 152 in the intervention group (46%) (195 [59%] men; 136 [41%] women; median age, 65 [IQR, 52-74] years) Most patients stayed in the acute medical unit (control, 138 [77%]; intervention 127 [84%]).

Baseline characteristics of the 331 patients in the control and intervention groups on the selected wards were comparable with regard to age (median years control, 65 [IQR, 52-74] years; intervention, 65 [IQR, 55-74] years), sex (control, 105 [59%] male; intervention, 90 [59%]), prehospital insomnia (no clinically significant insomnia), and physical and mental health status. Patients in the intervention group stayed less often on the surgical ward (control, 23 [13%]; intervention, 3 [2%]) and more often in fully occupied (3 other patients) rooms (control, 8 [5%]; intervention, 64 [42%]) (Table 1).

**Table 1.** Baseline Characteristics^a^

Characteristic	No. (%)	
	Control group (n = 179)	Intervention group (n = 152)
Age, median (IQR), y	65 (52-74)	65 (55-74)
Age, y		
<50	34 (19)	26 (17)
50-59	25 (14)	19 (13)
60-69	45 (25)	45 (30)
70-79	52 (29)	39 (26)
≥80	17 (10)	15 (10)
Missing	6 (3)	8 (5)
Sex		
Female	74 (41)	62 (41)
Male	105 (59)	90 (59)
Highest level of education		
Primary	11 (6)	8 (5)
Lower secondary vocational	21 (12)	15 (10)
Junior general secondary	21 (12)	19 (13)
Middle secondary vocational	46 (26)	41 (27)
Higher general secondary	17 (10)	12 (8)
Preuniversity	10 (6)	3 (2)
Higher vocational	24 (13)	30 (20)
University	24 (13)	16 (11)
Missing	5 (3)	8 (5)
Ward		
Acute medical unit	138 (77)	127 (84)
Medical unit (internal medicine/nephrology)	18 (10)	22 (14)
Surgical unit (vascular surgery/urology)	23 (13)	3 (2)
No. of other patients in room		
0	31 (17)	22 (15)
1	121 (68)	41 (27)
2	19 (11)	24 (16)
3	8 (5)	64 (42)
Missing	0	1 (1)
Undergone surgery in previous days	29 (16)	31 (20)
Physical and mental health status, median (IQR)		
Clinical Frailty Scale^b^	3 (2-4)	3 (3-4)
Charlson Comorbidity Index^c^	4 (2-6)	4 (2-6)
Obstructive sleep apnea syndrome	19 (11)	6 (4)
Prehospital insomnia, median (IQR)^d^	4 (1-11)	5 (1-12)
Absence	104 (58)	86 (57)
Subthreshold/threshold	36 (20)	33 (22)
Moderate	20 (11)	14 (9)
Severe	6 (3)	5 (3)
Missing	13 (7)	14 (9)

^a^ All data were collected after the first night of study participation.

^b^ Clinical Frailty Scale; level based on the clinical judgment of a patient’s frailty. Range 1 (very fit) to 9 (terminally ill, life expectancy <6 months).

^c^ Charlson Comorbidity Index. Predicting survival based on age and comorbidities. Range 0 to 34; higher scores indicate more severe comorbidities.

^d^ Insomnia Severity Index concerning sleep 30 days before hospital admission. Items are rated on a 5-point Likert scale with total scores between 0 and 28 points. Range 0 to 7 points indicates no clinically significant insomnia; 8 to 14 points, subthreshold insomnia; 15 to 21 points, moderate insomnia; and 22 to 28 points, severe insomnia.

### Sleep Quantity and Quality

The TST was extended by 40 minutes in the intervention group (median control, 6 hours and 5 minutes [IQR, 4 hours and 55 minutes to 7 hours and 4 minutes]; intervention, 6 hours and 45 minutes [IQR, 5 hours and 47 minutes to 7 hours and 39 minutes]; *P* < .001; Cohen *d* = 0.4). This difference was mainly due to a delayed final wake time of 30 minutes (median clock-time control, 6:30 am [IQR, 6:00-7:22 am]; intervention, 7:00 am [IQR, 6:30-7:30 am]; *P* < .001). No significant differences were found in sleep-onset latency (0 minutes), number and duration of nocturnal awakenings (sleep diary, 3 times; actigraphy, 12-13 times; median total, control, 1 hour and 7 minutes; intervention, 1 hour and 8 minutes) and daytime sleep duration (both, 1 hour and 30 minutes). Sleep quality did not differ significantly between the groups (median raw summary PROMIS score: control, 24 [IQR, 17-30.5]; standardized T score, 54.3 (SE, 2.5); intervention, 23 [IQR, 16-29]; T score 53.3 (SE 2.5); *P* = .54). No confounding or effect modification was found for age, sex, or number of other patients in the room (Table 2). Analysis of all included patients showed similar results. Subgroup analyses for patients staying in the acute medical unit showed larger statistically significant differences in TST of 44 minutes and final wake time of 45 minutes (eTable 3 in Supplement 2).

**Table 2.** Sleep Quantity and Quality^a^

Variable	Median (IQR)		Difference	*P* value
	Control	Intervention		
**Sleep quantity** ^b^				
No.	157	126		
Closing eyes to sleep time, clock time	10:00 pm (10:30 pm to 12:00 am)	10:00 pm (11:00 pm to 11:48 pm)	0	.21
Sleep-onset latency, duration, min^c^	0 (0-7)	0 (0-5)	0	.11
No. of awakenings				
Actigraphy	12 (9-18)	13 (8-19)	1	.82
Consensus Sleep Diary	3 (2-5)	3 (2-5)	0	.79
Median duration of each awakening, min	5 (3-7)	6 (4-7)	1	.34
Wake after sleep onset, duration^d^	1 h, 7 min (42 min to 1 h, 42 min)	1 h, 8 min (36 min to 1 h, 49 min)	1 min	.81
Final wake time, clock time				
Mean (SD)	6:30 am (1 h, 23 min)	6:58 am (1 h, 7 min)	28 min	.001
Median (IQR)	6:30 am (6:00 to 7:22 am)	7:00 am (6:30 to 7:30 am)	30 min	<.001
Planned sleep episode, duration^e^				
Mean (SD)	7 h, 24 min (1 h, 56 min)	8 h, 4 min (1 h, 56 min)	40 min	.005
Median (IQR)	7 h, 40 min (6 h, 30 min to 8 h, 30 min)	8 h (7 h to 9 h)	20 min	.007
Total sleep time, duration^f^				
Mean (SD)	5 h, 57 min (1 h, 46 min)	6 h, 36 min (1 h, 46 min)	39 min	.002
Median (IQR)	6 h, 5 min (4 h, 55 min to 7 h, 4 min)	6 h, 45 min (5 h, 47 min to 7 h, 39 min)	40 min	<.001
Sleep efficiency, %^g^	84 (77-90)	85 (78-91)	1	.28
Time attempting to sleep after final awakening, duration	1 h (30 min to 1 h, 30 min)	1 h (20 min to 1 h, 30 min)	0	.02
Daytime sleep, duration	1 h, 30 min (1 h to 2 h, 26 min)	1 h, 30 min (1 h to 2 h, 5 min)	0	.85
**Sleep quality (PROMIS sleep disturbance)**				
No.	178	150		
My sleep was restless (1, not at all to 5, very much)	3 (2-4)	3 (2-4)	0	.73
I was satisfied with my sleep (1, very much to 5, not at all)	3 (2-4)	3 (2-4)	0	.64
My sleep was refreshing (1, very much to 5, not at all)	4 (3-5)	4 (2-4.5)	0	.33
I had difficulty falling asleep (1, not at all to 5, very much)	2 (1-4)	2 (1-4)	0	.40
I had trouble staying asleep (1, not at all to 5, very much)	3 (2-4)	3 (2-4)	0	.70
I had trouble sleeping (1, not at all to 5, very much)	2 (1-4)	3 (1-4)	1	.74
I got enough sleep (1, very much to 5, not at all)	3 (2-4)	3 (2-4)	0	.43
My sleep quality was (1, very good to 5, very poor)	3 (2-4)	3 (2-4)	0	.72
Raw summary PROMIS score (8, very good sleep to 40, very poor sleep)	24 (17-30.5)	23 (16-29)	−1	.54
Standardized T score (SE)^h^	54.3 (2.5)	53.3 (2.5)	−1	.54

Abbreviation: PROMIS, Patient-Reported Outcomes Measurement Information System.

^a^ Analysis excluded patients who did not sleep at all (control, n = 2; intervention, n = 5; *P* = .17).

^b^ Closing eyes to sleep time, number of awakenings, final wake time, sleep duration, time attempting to sleep after final awakening and daytime sleep were obtained through sleep diaries, sleep onset latency, number of awakenings, median duration of awakenings, total sleep time, and sleep efficiency were analyzed using actigraphy.

^c^ Time it took to fall asleep after closing eyes to sleep.

^d^ Time spent awake after onset of sleep.

^e^ Time interval from closing eyes to sleep to final awakening.

^f^ Planned sleep episode minus time spent awake.

^g^ Total sleep time / planned sleep episode × 100.

^h^ Raw summery PROMIS scores correlate to standardized T scores, with a T score of 50 being the average score in the reference population (community dwelling adults [88%] and patients with sleeping disorders recruited from sleep medicine, general medicine, and psychiatric clinics [12%] living in the US).

### Patient-Reported Sleep-Disturbing Factors

Table 3 reports the most frequently mentioned causes of sleep-onset latency, nocturnal awakenings, and final awakening. Both nocturnal awakenings and final awakening in the control group were significantly more often caused by hospital staff (nocturnal awakenings due to noise of the hospital staff: control, 17%; intervention, 9%; *P* = .03; final awakening caused by hospital staff: control, 46%; intervention, 34%; *P* = .02). Patients in the intervention group more frequently reported nocturnal awakenings due to noise of other patients (control, 23%; intervention, 36%; *P* = .01) and concerns about disease (control, 8%; intervention, 15%; *P* = .04). No major differences were found in other patient-reported sleep-disturbing factors.

**Table 3.** Sleep-Disturbing Factors^a^

Variable	No. (%)		*P* value
	Control (n = 179)	Intervention (n = 152)	
**Sleep-onset latency**			
Noises of other patients	32 (18)	36 (24)	.19
Pain	32 (18)	26 (17)	.85
Noise from medical devices	29 (16)	24 (16)	.92
Uncomfortable sleeping position	26 (15)	20 (13)	.72
Light	24 (13)	24 (16)	.54
Concerns about disease	16 (9)	22 (14)	.12
Kept awake by hospital staff	21 (12)	21 (14)	.57
**Nocturnal awakenings**			
Toilet visits	92 (51)	64 (42)	.09
Noises of other patients	42 (23)	55 (36)	.01
Awakened by hospital staff	41 (23)	26 (17)	.19
Noises from medical devices	37 (21)	35 (23)	.61
Pain	37 (21)	32 (21)	.93
Noises of hospital staff	30 (17)	13 (9)	.03
Concerns about disease	14 (8)	23 (15)	.04
**Final awakening**			
Awakened by hospital staff	82 (46)	51 (34)	.02
Spontaneous	48 (27)	35 (23)	.43
Toilet visits	19 (11)	15 (10)	.82
Noises of other patients	14 (8)	21 (14)	.08
Noises of hospital staff	12 (7)	24 (16)	.008
Self-set alarm clock	1 (1)	6 (4)	.03
Noises from medical devices	7 (4)	14 (9)	.049

^a^ Patients were allowed to select more than 1 disturbing factor.

### Protocol Adherence and Use of Sleep Aids

During the intervention phase of the study, 100% of patients received a sleep folder from the researchers. All clinical lessons were delivered as planned, and the change in the morning routine interventions was successfully implemented. The change in morning routines was well received by the health care workers and continued to exist after the study had ended. Of all patients included in the intervention group, 39% reported the use of 1 or more sleep aids. Sleep masks were used by 16% (23 of 147) of patients and were perceived as beneficial by 70% (16 of 23) of the users. Earplugs were used by 12% (17 of 147) of patients and perceived as beneficial by 83% (14 of 17). All other sleep aids provided were used less frequently and had modest to no patient-reported sleep-enhancing outcomes (Table 4).

**Table 4.** Use of Sleep-Enhancing Interventions^a^

Variable	No. (%)		
	Used, helped	Used, did not help	Did not use
Sleep aids provided in the sleep folder			
Sleep mask	16 (11)	7 (5)	124 (84)
Earplugs	14 (10)	3 (2)	130 (88)
Aromatherapy (lavender oil)	7 (5)	8 (5)	132 (90)
Caffeine-free tea	3 (2)	2 (1)	142 (97)
Sleep music playlist (QR code)	1 (1)	1 (1)	145 (99)
Meditation (QR code)	1 (1)	1 (1)	145 (99)
Recommended in the sleep folder (Tips&Tricks)			
Asked to close door/curtain	27 (18)	5 (4)	115 (78)
Asked for extra blanket	23 (16)	3 (2)	121 (82)
Asked for extra pillow	9 (6)	3 (2)	135 (92)
Asked for extra socks	4 (3)	1 (1)	142 (97)
Avoided coffee after 3 pm after reading tips	3 (2)	3 (2)	141 (96)
Tried not to nap after 1 pm after reading tips	2 (1)	0	145 (99)
Got out of bed more after reading tips	1 (1)	0	146 (99)
Sleep aids on patient’s own initiative			
Medication	30 (20)	4 (3)	113 (77)
Sleep music (own playlist)	3 (2)	4 (3)	140 (95)
Meditation (own source)	1 (1)	2 (1)	144 (98)
Other^b^	2 (1)	0	145 (99)

Abbreviation: QR, quick response.

^a^ A total of 57 of 147 patients (39%) tried at least 1 intervention.

^b^ Followed advice to not hesitate or feel guilty to ask nurses for help during the night, move to another room, or watch television.

There was a major decrease in vital sign checks between 10:00 pm and 6:00 am (control, 54%; intervention, 11%; *P* < .001). No significant differences were found in the distribution of potential sleep-enhancing or sleep-disturbing medications between the groups (eTable 4 in Supplement 2).

### Clinical Outcomes

No major differences were found between the control and intervention groups in short-term clinical outcomes, such as depression, anxiety, pain, and Modified Early Warning Scores (eTable 4 in Supplement 2). In addition, there were no substantial differences between groups for other relevant outcomes, such as length of stay, incidence of intensive care unit admission, delirium, or hospital readmission (eTable 5 in Supplement 2).

## Discussion

In this study, implementation of simple nonpharmacologic interventions was associated with a 40- to 45-minute increase in the TST of inpatients. The largest increase was made by postponing morning medication and vital sign check rounds from the night to the morning shift. Feasibility studies conducted before 2003 showed medication times and sleeping patterns before and during admission were more closely aligned with postponement of morning rounds.^27,49^ Despite the recommendations by the authors of those studies to examine the association between changing morning logistics and patients’ sleep, to our knowledge, this was never executed until now. The possibility of improvement was to be expected, because many studies show final wake times in hospitalized patients of even before 6:00 am, with the main cause for these early awakenings being hospital staff.^50,51,52^

Despite the increase in sleep duration, no significant improvement in perceived sleep quality was found in this study. It is plausible that the experienced sleep quality is more affected by the number of nocturnal awakenings than the TST. Patients with substantially fragmented sleep are being stopped from cycling into deeper sleep stages essential to obtain fully restorative sleep.^53^

Ambient noise and light were among the most frequently mentioned sleep-disturbing factors in this study. In line with these findings, sleep masks and earplugs proved to be the best sleep-enhancing interventions as rated by patients (sleep masks, 70%; earplugs, 83%). However, these interventions were not associated with better sleep at the group level, because, of all patients, only 16% used sleep masks and 12% used earplugs. This finding is in line with earlier randomized controlled trials in which adherence rates of 60% to 65% were found, even with night nurses recommending that patients use the tools.^36,37,38^ Among the reasons for nonadherence were physical discomfort caused by eye masks or earplugs and the wish to be aware of the environment.^36,37^

The following recommendations for clinical practice can be made. First, postponement of early morning nursing rounds is recommended because the delays were associated with delayed final wake times and increased TST. These changes in morning routines were well received by the hospital staff and maintained after the study was completed. We therefore recommend this intervention because it is relatively simple and may have a potentially large benefit for inpatient sleep. Second, despite the fact that patients often stated they were disturbed by noise and light, interventions such as sleep masks and earplugs were unpopular. Although some individuals reported benefit, to improve the overall sleep of patients it seems reasonable to implement more systemic solutions.

Future research could focus on whether more comfortable sleeping masks and earplugs would increase popularity and whether there are alternatives to attenuate external stimuli at the patient level without completely shutting the patient off from the outside world (such as a dome designed to reduce exposure to sound and light).^54^

### Strengths and Limitations

The strength of this study lies in the mixture of medical and surgical patients and broad inclusion criteria allowing the results to be generalized across a substantial part of the general inpatient population. Recruitment 7 days per week during the same months with a 1-year difference for control and intervention patients avoided potential weekend and seasonal bias. A first-night effect (responsible for a decrease in sleep owing to not sleeping in one’s own bed) was minimized by including all patients after spending 1 night in the hospital.^55^ The interventions were developed in close consultation with patients and nurses, ensuring the relevance and feasibility in clinical practice.

The study has limitations. Owing to restrictions following the SARS-CoV-2 virus outbreak in February 2020, several wards were unable to continue study participation (they became a COVID-19 department, the patient population changed too much, or too many beds were closed owing to staff shortages). Fortunately, the study could continue in the wards where most patients were included until that time. Analyses on the data sets including and excluding the later excluded wards showed similar results, and we therefore assume that these decreased numbers did not greatly influence the results. Baseline characteristics were critically examined to rule out that the patient mix had changed owing to the pandemic. The only notable difference was the number of patients in a single room. Whereas during the intervention period most patients stayed in fully occupied rooms (with 3 other patients), most control patients shared their room with only 1 other individual. Previous studies showed that having more roommates is associated with worse sleep, suggesting that the difference in sleep found in the present study might be an underestimate of reality.^56,57^ Another consequence of the virus outbreak was the decrease in potential eligible patients. Despite not reaching the calculated study size, a relevant and significant increase in the primary outcome (TST) was found. Post hoc power analysis revealed a power of 87%. Moreover, our precalculated sample size was approximately 5 to 10 times higher than in similar studies.^33,34,35^ In addition, many patients lacked the capacity to consent owing to cognitive dysfunction or being continuously asleep during the day. Excessive daytime sleep and confusion may be manifestations of and predictors for poor sleep.^58,59,60^ Therefore, the poor TST found in this study might be better than the true median TST of inpatients.

## Conclusions

In this study, the implementation of simple nonpharmacologic interventions was associated with a 40- to 45-minute increase in TST of hospitalized patients. This increase was mainly due to a 30- to 45-minute delay in final wake time. Postponement of early morning nursing rounds seems to be a feasible, useful, and sustainable sleep-enhancing intervention.

## Notes

Supplement 1.
Trial Protocol

Supplement 2. **eFigure 1.** Sleep Folder With Tips&Tricks, QR-codes, and Theoretical Foundation **eFigure 2.** One of the Posters as Developed by the Nurses **eTable 1.** Sleep Hygiene Recommendations for Patients **eTable 2.** Sleep Hygiene Instructions for Nurses Based on Dreher’s Conceptual Model **eTable 3.** Sleep Quantity and Quality Subgroups **eTable 4.** Factors Bidirectionally Associated With Sleep **eTable 5.** Clinical Outcomes
