# Supplementary material for: Evaluation of Nonpharmacologic Interventions and Sleep Outcomes in Hospitalized Medical and Surgical Patients: A Nonrandomized Controlled Trial
Source: JAMA Netw Open. 2022 Sep 21;5(9):e2232623. doi: 10.1001/jamanetworkopen.2022.32623 (PMC9494194; doi:10.1001/jamanetworkopen.2022.32623)
Supplement: Supplement 1. — Trial Protocol [file jamanetwopen-e2232623-s001.pdf]

Research protocol

General information

**Title**	Improving quality and quantity of sleep in Hospitalized Patients
**Date**	13 April 2019
**Version number**	Version 1
**Applicants**	Eva van den Ende, MD, PhD student acute internal medicine
**Coordinating researchers**	Drs. Eva van den Ende<br>(Amsterdam UMC, location VUmc, section acute medicine,<br>Department of internal medicine)<br>e.vandenende@amsterdamumc.nl
**Principal investigator**	Prof. Dr. P.W.B. Nanayakkara (Amsterdam UMC, location VUmc, section acute medicine, Department of internal medicine, De Boelelaan 1117, 1081 HV, Amsterdam. Room number 1 D 053<br>p.nanayakkara@amsterdamumc.nl
**Coordinating site**	Amsterdam UMC, location VUmc

Research information

**Rationale**	It is known that inadequate quantity and quality of sleep is negatively associated with health and wellbeing of the sick and the healthy (1-4). In patients, the lack of sleep is associated with physiological disturbances such as respiratory and metabolic changes, which may result in poor healing (5-10). In a recently performed single-day, multicentre, observational study coordinated by section acute medicine of VUmc, 2005 patients in 39 hospitals were asked about the sleep quality and quantity of the previous night and their habitual sleep at home a month before admission. This study, recently published in JAMA internal medicine, found that both the quality and quantity of sleep of in-patients were negatively affected, due to

	many hospital-related factors such as noise of other patients, noise of hospital equipment, pain and toilet visits (11).<br>A few studies on the effectiveness of interventions to improve the quality of sleep have been conducted in the past, mainly on the Intensive Care (IC) Unit. Interventions such as providing patients with earplugs and sleeping masks, dimming the hallway lights, involving patients in what they need to improve their sleep and implementing sleep hygiene education for nurses have been found to improve both sleep quantity and quality in the IC units (12-15). However, no large scale multicentre studies have been performed on the potential effectiveness of these interventions in regular wards. Therefore, the aim of this study is to implement interventions (such as care-clustering, better mattresses/pillows/blankets, the distribution of earplugs and sleep masks, sleep education to patients, nurses and doctors, adjustment of the hospital rhythm (15)) and to measure whether these interventions have a positive effect on the (experienced and absolute) quality and quantity of sleep in regular hospital wards.
**Aim**	Primary outcome measure:<br>- The effect of implemented sleep interventions on the quantity of sleep.<br><br>Secondary outcome measures:<br>- To explore the potential effects of the interventions on other sleep outcomes, the use of sleep medication, length of stay (including discharge diagnosis: homewards/palliative policy/rehabilitation etc), incidence of delirium and incidence of ICU admissions from the clinical wards.
**Design**	Before and after study.<br>- 8 months baseline measurement<br>- 3 months preparation of interventions<br>- 8 months introduction of intervention and measurement
**Population**	All ≥18-year-old medical patients admitted for at least one night to one of the participating wards.

**Inclusion criteria**	- >18 years of age<br>- Admitted to Acute Medical Units (AOA), internal medicine/nephrology wards or vascular diseases/urology unit (with the possibility to extend the research to other wards)<br>- Able to give informed consent<br>- Admission to the ward admitted before 3:00 AM that day
**Exclusion criteria**	- <18 years of age<br>- Not able to give informed consent (i.e. severe illness, not capable of speaking Dutch, cognitive dysfunction)<br>- Admission to the ward after 3:00 AM that day
**Sample size**	Similar research has, by our knowing, has only been done at the ICU. Patients at the ICU will have different reasons for sleep disruption and therefore we cannot use these data to calculate a sample size. Based on our previous research, we aim to include 286 patients in each group.
**Recruitment**	Every workday a researcher will identify together with the treating doctor or intern all potential eligible patients. They will be asked to participate in this project. When patients agree to participate and sign the informed consent document, the patient will receive a sleep registration bracelet for two nights. The following two mornings one of the researchers will visit the patient before 12:00 A.M. for a short interview. The questionnaires will contain questions concerning sleep quality and quantity of the prior night and a month before admission. All data will be registered in the protected Castor database. The results of the sleep registration bracelets will be coded with the record number generated by Castor. The bracelets will be re-used after sterilisation in the hospital.
**Intervention**	<u>Interventions:</u><br>Interventions will be developed in close consultation with clinicians and patients after the baseline data has been collected.<br>Interventions could focus on:<br>- Better (comfortable) mattresses/pillows/blankets<br>- Earplugs, sleep masks<br>- Door stops, light strips in hall way, dimmable lights

	- Posters to increase awareness with medical team<br>- Sleep education patients (information flyer, inform about possibility to bring own pillow from home, etcetera)<br>- Sleep hygiene training nurses and doctors (awareness, “soft” shoes, use little light, if possible place restless patients in single room, during day time: stimulate patients to leave their beds, open curtains and blinds, discourage afternoon naps and caffeine after 15:00 PM, avoid giving I.V. drips overnight if possible or diuretics in the afternoon if not necessary with the aim of reducing nightly toilet visits, take only vitals when necessary)<br>- Adjust hospital rhythm (only measure vital signs in morning if indicated, wash patients after morning rounds)<br>- Exploring the possibility to use venflon cannulas instead of drips to increase freedom in movement.<br>- ward-specific plan of improvement
**Study endpoints**	<u>Measurements after first to fifth night:</u><br>- Short questionnaire (see Appendix F1, F2)<br>o Sleep quality (Patient-Reported Outcomes Measurement Information System (PROMIS™) - based questions (11, 16))<br>o Sleep quantity (Consensus Sleep Diary (CSD)) (11, 17)<br>- Measured sleep quantity (with sleep tracker (e.g. Actigraph or FitBit)<br><br><u>After 30 days (from medical record):</u><br>- Length of stay (including discharge diagnosis: homewards/palliative policy/rehabilitation etc)<br>- Incidence of delirium<br>- Incidence of unplanned ICU admissions
**Other study parameters**	Age, sex, number of patients in room, use of sleep medication and other sleep promoting interventions, potential sleep disturbing

	factors (i.e. use of diuretics, drips), MEWS scores, Clinical Frailty index, Charlson Comorbidity Index , clinical diagnosis)
**Statistical analyses**	Analysis will mainly focus on the first night collected data.<br>Descriptive data-analyses will provide mean, median, 95% confidence intervals (CI) and inter-quartile ranges (IQR) for continuous variables and percentages for categorical variables.<br>Mean difference scores in sleep quantity (Actigraphy) and quality (PROMIS total score) will be compared between groups (intervention- and control group). Confounding and effect modification were assessed using multiple linear regression analysis.<br>Normality will be checked by visual inspection of histograms.<br>p-values of <0.05 will be considered statistically significant. Analysis will be performed using Statistical Package for Social Sciences for Windows (SPSS).
**Burden for patients**	Sleeping with a wrist band<br>Filling out questionnaires up to five times (time investment 10 minutes)
**Potential risk for patients**	No risks applicable
**Advantages of participating in study**	At this moment there are no advantages for the patient to participating in this study. In the future we hope to increase awareness and on the basis of the results of the interventions improve the quality and quantity of sleep in hospitalized patients in the Netherlands.
**Disadvantages of participating in study**	It takes a few minutes to fill out the questionnaire
**Patient reimbursement**	No compensation applicable
**Administrative aspects**	No traceable patient data will be collected. Data will be saved in a secured Castor database. Data will be analysed in the Amsterdam UMC, Location VUmc.
**Publication policy and amendments**	The goal is to publish the results in a medical scientific journal and present during a scientific conference.
**Other**	
**References**	1. Alvarez GG, Ayas NT. The impact of daily sleep duration on health: a review of the literature. Prog Cardiovasc Nurs. 2004;19(2):56-9.<br><br>2. Gamaldo CE, Shaikh AK, McArthur JC. The sleep-immunity relationship. Neurol Clin. 2012;30(4):1313-43.

3. Gottlieb DJ, Punjabi NM, Newman AB, Resnick HE, Redline S, Baldwin CM, et al. Association of sleep time with diabetes mellitus and impaired glucose tolerance. Arch Intern Med. 2005;165(8):863-7.

4. Hoevenaar-Blom MP, Spijkerman AM, Kromhout D, van den Berg JF, Verschuren WM. Sleep duration and sleep quality in relation to 12-year cardiovascular disease incidence: the MORGEN study. Sleep. 2011;34(11):1487-92.

5. Cooper KR, Phillips BA. Effect of short-term sleep loss on breathing. J Appl Physiol Respir Environ Exerc Physiol. 1982;53(4):855-8.

6. Irwin MR, Wang M, Campomayor CO, Collado-Hidalgo A, Cole S. Sleep deprivation and activation of morning levels of cellular and genomic markers of inflammation. Arch Intern Med. 2006;166(16):1756-62.

7. Kahn-Greene ET, Killgore DB, Kamimori GH, Balkin TJ, Killgore WD. The effects of sleep deprivation on symptoms of psychopathology in healthy adults. Sleep Med. 2007;8(3):215-21.

8. Knutson KL, Spiegel K, Penev P, Van Cauter E. The metabolic consequences of sleep deprivation. Sleep Med Rev. 2007;11(3):163-78.

9. Mesarwi O, Polak J, Jun J, Polotsky VY. Sleep disorders and the development of insulin resistance and obesity. Endocrinol Metab Clin North Am. 2013;42(3):617-34.

10. Morselli LL, Guyon A, Spiegel K. Sleep and metabolic function. Pflugers Arch. 2012;463(1):139-60.

11. Wesselius HM, van den Ende ES, Alsma J, Ter Maaten JC, Schuit SCE, Stassen PM, et al. Quality and Quantity of Sleep and Factors Associated With Sleep Disturbance in Hospitalized Patients. JAMA Intern Med. 2018;178(9):1201-8.

12. Faraklas I, Holt B, Tran S, Lin H, Saffle J, Cochran A. Impact of a nursing-driven sleep hygiene protocol on sleep quality. J Burn Care Res. 2013;34(2):249-54.

13. Gathecha E, Rios R, Buenaver LF, Landis R, Howell E, Wright S. Pilot study aiming to support sleep quality and duration during hospitalizations. J Hosp Med. 2016;11(7):467-72.

14. Hu RF, Jiang XY, Chen J, Zeng Z, Chen XY, Li Y, et al. Non-pharmacological interventions for sleep promotion in the intensive care unit. Cochrane Database Syst Rev. 2015(10):CD008808.

15. Li SY, Wang TJ, Vivienne Wu SF, Liang SY, Tung HH. Efficacy of controlling night-time noise and activities to improve patients' sleep quality in a surgical intensive care unit. J Clin Nurs. 2011;20(3-4):396-407.

16. Cella D, Riley W, Stone A, Rothrock N, Reeve B, Yount S, et al. The Patient-Reported Outcomes Measurement Information System (PROMIS) developed and tested its first wave of adult self-reported

	health outcome item banks: 2005-2008. J Clin Epidemiol. 2010;63(11):1179-94.<br><br>17.Carney CE, Buysse DJ, Ancoli-Israel S, Edinger JD, Krystal AD, Lichstein KL, et al. The consensus sleep diary: standardizing prospective sleep self-monitoring. Sleep. 2012;35(2):287-302.
