# Supplementary material for: Evaluation of Nonpharmacologic Interventions and Sleep Outcomes in Hospitalized Medical and Surgical Patients: A Nonrandomized Controlled Trial
Source: JAMA Netw Open. 2022 Sep 21;5(9):e2232623. doi: 10.1001/jamanetworkopen.2022.32623 (PMC9494194; doi:10.1001/jamanetworkopen.2022.32623)
Supplement: Supplement 2. — eFigure 1. Sleep Folder With Tips&Tricks, QR-codes, and Theoretical Foundation eFigure 2. One of the Posters as Developed by the Nurses eTable 1. Sleep Hygiene Recommendations for Patients eTable 2. Sleep Hygiene Instructions for Nurses Based on Dreher’s Conceptual Model eTable 3. Sleep Quantity and Quality Subgroups eTable 4. Factors Bidirectionally Associated With Sleep eTable 5. Clinical Outcomes [file jamanetwopen-e2232623-s002.pdf]

Supplementary Online Content

van den Ende ES, Merten H, Van der Roest L, et al. Evaluation of nonpharmacologic interventions and sleep outcomes in hospitalized medical and surgical patients: a nonrandomized controlled trial. *JAMA Netw Open*. 2022;5(9):e2232623. doi:10.1001/jamanetworkopen.2022.32623

eFigure 1. Sleep Folder With Tips&Tricks, QR-codes, and Theoretical Foundation

eFigure 2. One of the Posters as Developed by the Nurses

eTable 1. Sleep Hygiene Recommendations for Patients

eTable 2. Sleep Hygiene Instructions for Nurses Based on Dreher's Conceptual Model

eTable 3. Sleep Quantity and Quality Subgroups

eTable 4. Factors Bidirectionally Associated With Sleep

eTable 5. Clinical Outcomes

This supplementary material has been provided by the authors to give readers additional information about their work.

© 2022 van den Ende ES et al. *JAMA Network Open.*

Amsterdam UMC
Universitair Medische Centra

Informatiefolder

Slaap als medicijn zonder bijwerkingen

Ondanks dat u niet in uw eigen bed slaapt, willen wij er alles aan doen u ook hier zo goed mogelijk te laten slapen. Waarom?

Uit onderzoek blijkt dat een goede nachtrust belangrijk is voor:

- Beter (en sneller) herstel
- Een betere afweer
- Minder stress
- Minder pijn
- Minder angst en negatieve gedachten
- En nog veel meer * …

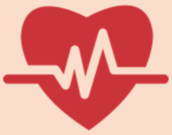

* Optimaliseert de stofwisseling, herstelt de hormoonbalans en verbetert het geheugen.

Hoe kan ik beter slapen? Kijk daarvoor op pagina 2

Tips & Tricks

Wat kunt u zelf doen om uw slaap te verbeteren?

- Uw normale bedtijd ritueel aanhouden
  - Bijv. tandenpoetsen, douchen en/of een boek lezen.
- Aan de bel trekken bij klachten, ook 's nachts!
  - Bij bijv. misselijkheid, pijn, honger, benauwdheid en/of slecht comfort.
- Probeer overdag uit bed te komen*
- Probeer geen middagdutjes te doen na 13.00 uur*

Maak eventueel gebruik van hulpmiddelen:

- Oordopjes / slaapmasker
- Een extra kussen, deken en/of sokken
  - U kunt hierom vragen bij de verpleegkundigen.
- Probeer geen koffie te drinken na 15.00 uur *
  - Probeer bijv. de slaapthee uit dit pakket (vraag bij de koffieronde van 20.00 om heet water).
- Sluit de kamerdeur of gordijn indien mogelijk
  - Vraag dit aan de verpleegkundigen
- Aromatherapie*
  - Leg het doekje lavendelolie naast u op uw kussen
- Ontspannende muziek/ bodyscan*
  - Scan dan de QR code

* Hoe zit dat dan? Op de volgende pagina kunt u vinden waarom deze adviezen bijdragen aan een goede nachtrust.

Waarom werken deze adviezen?

Waarom je moe wordt: Wanneer je wakker bent stapelt de chemische stof adenosine zich langzaam op in je hersenen. Hoe meer adenosine, hoe vermoeider je je voelt. Je hebt een hoge dosis nodig om in slaap te vallen. Als je slaapt neemt de concentratie adenosine weer af. Je hersenen spoelen als het ware schoon.

Cafeïne lijkt voor de hersenen op adenosine en bindt zich aan de adenosine-receptoren in de hersenen. Alleen omdat het geen adenosine is, word je er niet moe van. Nu zijn alle receptoren bezet met cafeïne en kan adenosine niet langer binden. Daarnaast stimuleert cafeïne de aanmaak van adrenaline (het vecht-vlucht hormoon dat zorgt voor een verhoogde hartslag en bloeddruk). En dopamine (wat het verslavend maakt). Kortom, je wordt alerter en krijgt meer energie.

Zo kunt u zich misschien voorstellen dat een kop koffie vlak voor het slapen gaan zorgt voor een onrustig gevoel. De kwaliteit van de nachtrust gaat hiermee omlaag. Je valt slecht in slaap, of wordt vaak wakker. Na 4 uur is pas de helft van het effect van een kopje koffie verdwenen. Wanneer je om 15.00 de laatste kop drinkt, is het effect van cafeïne pas na 23.00 uur verdwenen.

Overigens gelden deze effecten niet alleen voor koffie. 3 koppen (groene/zwarte) thee of een halve reep pure chocolade staan gelijk aan 1 kop koffie.

Je lichaam kent globaal twee standen: als het sympathische systeem wordt geprikkeld sta je in de actieve modus: je lichaam is klaar om te vechten of te vluchten. Je parasympatische systeem zorgt dat je lichaam in rust is. Dit is nodig om lekker te slapen.

Rustige muziek heeft een bewezen direct effect op het parasympatisch zenuwstelsel. Hierdoor komt je lichaam in een ontspannen modus. Verschillende studies hebben aangetoond dat mensen die naar rustgevende muziek luisteren voor het slapen gaan sneller in slaap vallen, minder tussendoor wakker worden en langer en lekkerder slapen (5, 6).

Heeft u een koptelefoon bij u? Scan de onderstaande QR-codes met de camera van uw smartphone voor rustgevende muziek of luister naar rustige muziek die u zelf fijn vindt.

Jazz | Klassiek

© 2022 van den Ende ES et al. *JAMA Network Open.*

Overdag slapen werkt 's nachts averechts. Doordat je kort slaapt verdwijnt de adenosine uit je hersenen en neemt de druk om weer te gaan slapen af. Je hebt een hoge slaapdruk nodig om s 'avonds in slaap te kunnen vallen. Daarom wordt geadviseerd na 15:00 uur niet meer te gaan slapen. Studies wijzen ook uit dat dagelijkse **beweging** zorgt voor een betere slaapkwaliteit. Door de activiteit van je spieren overdag, kan je lichaam 's nachts beter ontspannen (1).

Er is veel onderzoek gedaan naar het effect van slaapmedicatie en daaruit blijkt dat er op lange termijn veel bijwerkingen zijn. Daarom worden niet-medicamenteuze hulpmiddelen geadviseerd. Bijvoorbeeld **aromatherapie**. Veruit de meest onderzochte aromatherapie op het gebied van slaap is lavendelolie. Lavendelolie heeft kalmerende eigenschappen als het wordt ingeademd. Meerdere klinische studies hebben bewezen dat lavendelolie een veilige en effectieve manier is om sneller in te slaap te vallen en minder wakker te worden (2).

Wilt u uitproberen of dit ook voor u werkt?
In dit mapje vindt u een doekje met lavendelolie.
Leg dit uitgepakt naast u op uw hoofdkussen en sluit uw ogen.

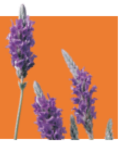

Piekeren is een vorm van stress. Stress is in de natuur een gezonde reactie om een korte periode veel energie vrij te maken om te overleven in een bedreigende situatie. Hierbij worden stresshormonen (adrenaline en cortisol) uitgescheiden door je bijnier en blijf je helder en alert. In het ziekenhuis is het echter niet vaak nodig om 's nachts actief te zijn, en houdt het je meestal voor niets uit je slaap.

Voor sommige mensen helpt het om hun **gedachten op papier te zetten** zodat ze niet bang meer zijn het te vergeten en het voor nu los kunnen laten. Voor andere mensen helpt het beter om gedachten te verzetten doormiddel van **visualisaties**, een (geleide) **meditatie**, **bodyscan** of **mindfulness** (3, 4).

Heeft u een koptelefoon bij u? Scan de onderstaande QR-codes met de camera van uw smartphone voor, visualisaties, geleide meditatie, of een bodyscan.*

Download in de App Store | Ontdek het op Google Play

**Disclaimer: Deze app van de zorgverzekering biedt een programma aan van 5 weken. Kies een aantal oefeningen uit die u aanspreekt en past bij uw situatie (bijv: ademhalingsoefeningen, (korte) bodyscan, geleide meditatie, maak kennis met visualisatie).*

De nummertjes in de tekst verwijzen naar wetenschappelijk onderzoek.

1. Yang PY, Ho KH, Chen HC, Chien MY. Exercise training improves sleep quality in middle-aged and older adults with sleep problems: a systematic review. *J Physiother*. 2012;58(3):157-163.
2. Lillehei AS, Halcon LL, Savik K, Reis R. Effect of Inhaled Lavender and Sleep Hygiene on Self-Reported Sleep Issues: A Randomized Controlled Trial. J Altern Complement Med. 2015;21(7):430-8.
3. Black DS, O'Reilly GA, Olmstead R, Breen EC, Irwin MR. Mindfulness meditation and improvement in sleep quality and daytime impairment among older adults with sleep disturbances: a randomized clinical trial. JAMA Intern Med. 2015;175(4):494-501.
4. Goyal M, Singh S, Sibinga EM, Gould NF, Rowland-Seymour A, Sharma R, et al. Meditation programs for psychological stress and well-being: a systematic review and meta-analysis. JAMA Intern Med. 2014;174(3):357-68.
5. Lai HL, Good M. Music improves sleep quality in older adults. J Adv Nurs. 2005;49(3):234-44.
6. Wang CF, Sun YL, Zang HX. Music therapy improves sleep quality in acute and chronic sleep disorders: a meta-analysis of 10 randomized studies. Int J Nurs Stud. 2014;51(1):51-62.

En er is nog veel meer bewijs dat niet-medicamenteuze slaaptherapiën voor veel mensen werken.

Amsterdam UMC
Universitair Medische Centra

Nogmaals bedankt voor het meedoen aan het onderzoek.

Bij vragen kunt u terecht bij de verpleegkundigen of u kunt contact opnemen met Drs. Eva van den Ende, uitvoerend arts-onderzoeker.

E: e.vandenende@amsterdamumc.nl

Wij wensen u een goede nachtrust toe!

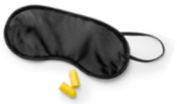

eFigure 1. Sleep Folder with Tips&Tricks, QR-codes and Theoretical Foundation

© 2022 van den Ende ES et al. *JAMA Network Open.*

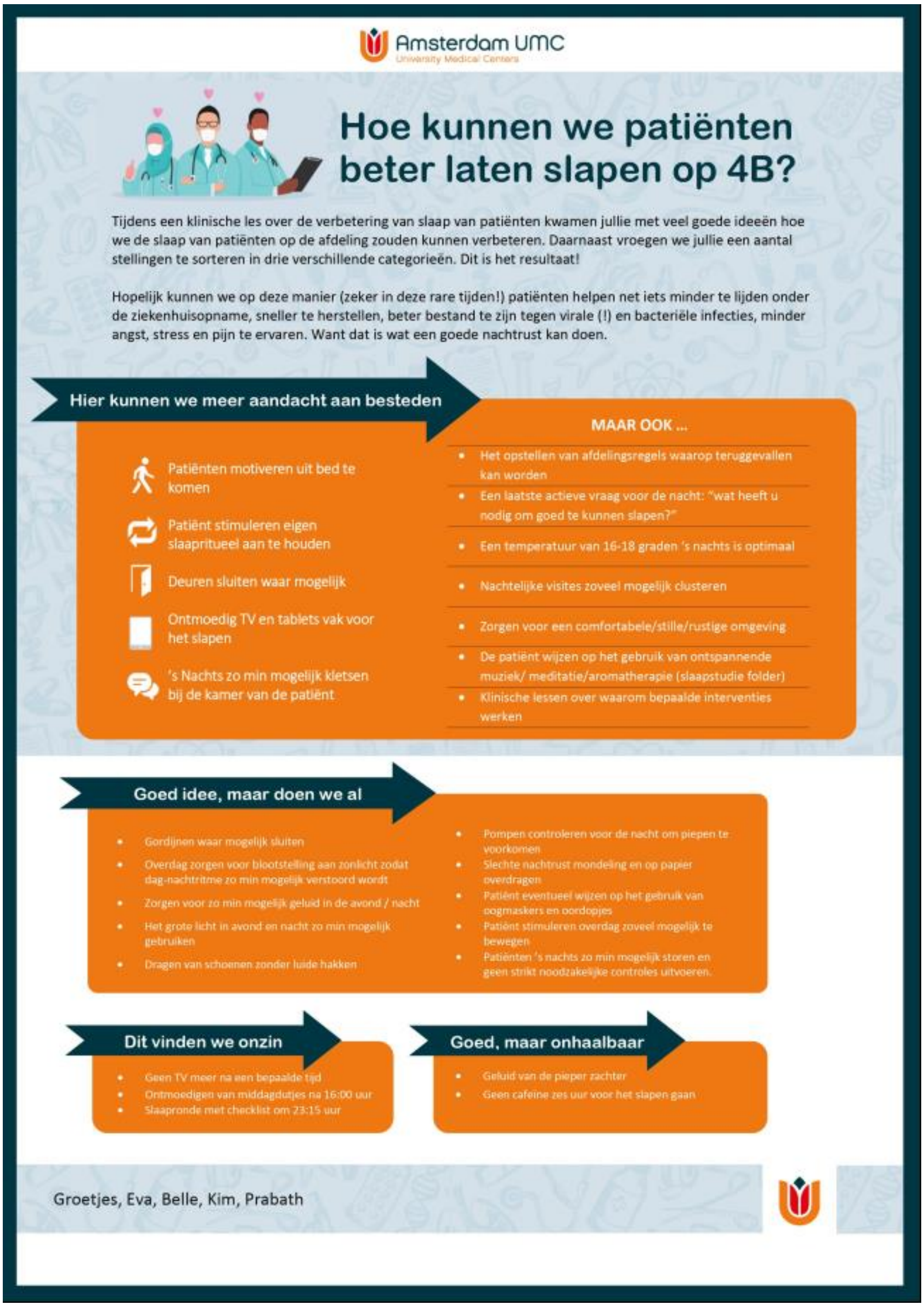

eFigure 2. One of the posters as developed by the nurses

The interventions designed by the nurses during the brainstorm session supplemented with the interventions proposed by the researchers were presented to the nurses. The nurses were asked to divide all interventions into several categories; things that could indeed be improved on this specific ward and would benefit patients' sleep, interventions that are useful but are already being applied in a reasonable manner, and interventions that are not considered to be useful or feasible. The poster showing the results of the workshop were hung in visible places on the ward.

eTable 1. Sleep hygiene recommendations for patients

**Physiological factors**		
	-	Tell your nurse before or during the night in case of physical discomfort (e.g. pain, sickness, dyspnea, hunger)
	-	Try not to consume caffeine after 3:00 pm (e.g. cola, root beer, iced tea, coffee)
	-	Try to avoid drinking >4 glasses of water one hour before sleeping
**Behavioural arousal**		
	-	Try to avoid activities that make you feel awake (watching TV, talking on the telephone)
	-	Listen to relaxing music / Audiotape guided imagery / read a book
**Cognitive and emotional factors**		
	-	Try to minimize thinking about things that need to be done at bedtime (e.g. by writing down things you don't want to forget/ want to ask your doctor)
**Sleep environment and sleep stability**		
	-	(Ask for) dim(med) lights after 10:00 pm / use eye mask
	-	Ask for adjustment of room temperature / extra blanket / extra pillows / socks
	-	Ask (nurse) for silence (if personnel or other patients bother you) after 10:00pm / Use earplugs
**Daytime sleep**		
	-	Try to avoid napping after 6:00 pm
**Substance use**		
	-	Tell your nurse before the night if you are used to use sleep medication at home
**Bedtime routine**		
	-	Try as much as possible to stick to your usual bedtime routine (e.g. bathing, brushing teeth, reading)

© 2022 van den Ende ES et al. *JAMA Network Open.*

eTable 2. Sleep hygiene instructions nurses based on Dreher's conceptual model

**Retiring phase (from preparation till onset of sleep):**		
	-	Discourage activities that make the patient feel awake (watching TV, talking on the telephone)
	-	Offer help with final restroom usage
	-	Offer warm milk or tea (without caffeine)
	-	Offer a warm/ extra blanket
	-	Adjust room temperature and dim lights
	-	Try to recognize strong emotions (e.g. sadness, anxiety, anger, excitement) at nighttime
	-	Evaluate physical discomfort before the night (e.g. pain, sickness, dyspnea, hunger)
	-	Check whether the patient is using sleep medication at home
	-	Help the patient as much as possible to stick to his/her usual bedtime routine (e.g. bathing, brushing teeth)
	-	Adjust room temperature to 16-18 degrees Celsius
**Resting phase (until first awakening):**		
	-	Close doors (or partially closing doors in case of close monitoring)
	-	Cluster visits (for single patients and for patients in the same room)
	-	Only take vitals when necessary
	-	Lower volumes of phones and alarms (if possible)
	-	Speak in low voices after 10:00pm (extra attention during change of shifts), avoid conversations in hallways.
	-	Use window blinds
	-	Dim lights and use little light when entering a patients' room
**Rising phase (until full arousal):**		
	-	Stimulate patients to leave their beds
	-	Open curtains and blinds
	-	discourage afternoon naps and caffeine after 15:00 PM
**In general:**		
	-	If possible place restless patients in single room
	-	Try to wear "soft" shoes
	-	Avoid administering I.V. drips overnight were possible
	-	Avoid administration of diuretics during evening and nights were possible
	-	Consider removing CAD before the night

© 2022 van den Ende ES et al. *JAMA Network Open.*

eTable 3. Sleep Quantity and Quality subgroups

	Control group			Intervention group		Difference (p-value) Intervention - Control					
	All wards (n=192)	Selected wards (n=157)	AMU (n=122)	All / selected wards (n=126)	AMU (n=104)	All wards		Selected wards		AMU	
**Sleep Quantity** (Actigraphy, Sleep Diary)											
**Closing Eyes to Sleep Time** Median [IQR]	23:00 [22:26 – 23:52]	23:00 [22:30 – 00:00]	23:00 [22:30 – 00:00]	23:00 [22:00-23:48]	23:00 [22:00-23:52]	00:00	0.255	00:00	0.212	00:00	0.131
**Sleep Onset Latency**[1] Median [IQR]	00:00 [00:00-00:06]	00:00 [00:00-00:07]	00:00 [00:00-00:06]	00:00 [00:00-00:05]	00:00 [00:00-00:05]	00:00	0.163	00:00	0.111	00:00	0.431
**Number of Awakenings,** No.<br>Actigraphy, Median [IQR]<br>CSD, Median [IQR]	12 [9-18]<br>3 [2-5]	12 [9-18]<br>3 [2-5]	12 [8-17]<br>3 [2-5]	13 [8-19]<br>3 [2-5]	13 [8-19]<br>3 [2-5]	1<br>0	0.614<br>0.796	1<br>0	0.815<br>0.791	1<br>0	0.622<br>0.536
**Median Duration of Each Awakening** Median [IQR]	00:05 [00:04-00:07]	00:05 [00:03-00:07]	00:05 [00:04-00:07]	00:06 [00:04-00:07]	00:06 [00:04-00:08]	00:01	0.261	00:01	0.337	00:01	0.108
**Wake After Sleep Onset**[2] Median [IQR]	01:06 [00:41-01:37]	01:07 [00:42-01:42]	01:07 [00:39-01:33]	01:08 [00:36-01:49]	01:11 [00:41-01:42]	00:02	0.557	00:01	0.813	00:03	0.479
**Final Wake Time**<br>Mean (SD)<br>Median [IQR]	06:27 (01:24)<br>06:30 [06:00-07:15]	06:30 (01:23)<br>06:30 [06:00-07:22]	06:28 (01:13)<br>06:15 [06:00-07:00]	06:58 (01:07)<br>07:00 [06:30-07:30]	06:55 (01:04)<br>07:00 [06:30-07:30]	31:00<br>30:00	0.001<br><0.001	28:00<br>30:00	0.001<br><0.001	27:00<br>45:00	0.002<br><0.001
**Planned sleep episode**[3]<br>Mean (SD)<br>Median [IQR]	07:20 (01:59)<br>07:35 [375-510]	07:24 (01:56)<br>07:40 [390-510]	07:14 (01:57)<br>07:30 [373-510]	08:04 (01:56)<br>08:00 [07:00-09:00]	08:03 (01:48)<br>08:03 [07:00-09:00]	00:44<br>00:25	0.001<br>0.003	00:40<br>00:20	0.005<br>0.007	00:49<br>00:33	0.001<br>0.002
**Total Sleep Time**[4]<br>Mean (SD)<br>Median [IQR]	05:56 (01:49)<br>06:05 [04:54-07:07]	05:57 (01:46)<br>06:05 [04:55-07:04]	05:50 (01:43)<br>06:01 [04:42-06:52]	06:36 (01:46)<br>06:45 [05:47-07:39]	06:32 (01:46)<br>06:45 [05:48-07:39]	00:40<br>00:40	0.001<br><0.001	00:39<br>00:40	0.002<br><0.001	00:42<br>00:44	0.003<br><0.001
**Sleep Efficiency**[5], % Median [IQR]	84 [77-90]	84 [77-90]	84 [78-90]	85 [78-91]	85 [77-91]	1	0.329	1	0.276	1	0.624
**Time Attempting to Sleep After Final Awakening** Median [IQR]	00:60 [00:30-01:38]	00:60 [00:30-01:30]	00:60 [00:30-01:35]	00:60 [00:20-01:30]	00:53 [00:20-01:30]	00:00	0.096	00:00	0.022	- 00:07	0.035
**Daytime Sleep** Median [IQR]	01:30 [01:00-02:30]	01:30 [01:00-02:26]	01:30 [01:00-02:30]	01:30 [01:00-02:05]	01:30 [01:00-02:01]	00:00	0.946	00:00	0.853	00:00	0.886
**Sleep Quality** (PROMIS Sleep Disturbance)											
**Raw summary PROMIS score**<br>Very good sleep quality (8) - Very poor sleep quality (40)	24 [17-31]	24 [17-31]	24 [17-29]	23 [16-29]	24 [16-30]	-1	0.315	-1	0.514	0	0.662

© 2022 van den Ende ES et al. *JAMA Network Open.*

Abbreviations: IQR, interquartile range; min, minutes; No., number; PROMIS, Patient-Reported Outcomes Measurement Information System (PROMIS™) Sleep Disturbance (SD)
All Sleep Quantity data are presented as Median hours:minutes unless indicated otherwise. Means (SD) are provided for data with normal distributions. Analysis exclude patients who did not sleep at all (control all wards 2, selected wards 2, AMU 0, intervention all/selected wards 5, AMU 5, in both datasets).
Closing Eyes to Sleep Time, Number of awakenings, Final Wake Time, Sleep Duration, Time Attempting to Sleep After Final Awakening and Daytime Sleep were obtained through Sleep Diaries. Sleep Onset Latency, Number of Awakenings, Median duration of Awakenings, Total Sleep Time and Sleep Efficiency were analyzed using Actigraphy.

[1]Time it took to fall asleep after closing eyes to sleep. [2]Time spent awake after onset of sleep. [3]Time interval from "Closing eyes to sleep" to "final awakening". [4]Planned sleep episode minus time spent awake. [5]Total Sleep Time divided by Planned sleep episode (*100).

© 2022 van den Ende ES et al. *JAMA Network Open.*

	Characteristics	Control group (n=173)	Intervention group (n=146)	P-value
**eTable 4. Factors bidirectionally associated with sleep**				
**Potential sleep-enhancing factors,** n (%)				
	Benzodiazepine use	24 (14)	17 (12)	0.616
	Melatonin use	2 (1)	0 (0)	0.502
	Opioid use	43 (25)	42 (29)	0.448
	Antipsychotic use	2 (1)	3 (2)	0.663
	Tricyclic antidepressant (TCA) use	8 (5)	4 (3)	0.557
	Antiepileptic drug use	12 (7)	6 (4)	.0334
	Antihistamine use	4 (2)	1 (1)	0.380
**Potential sleep-disturbing factors,** n (%)				
	Corticosteroid use (systemic)	11 (6)	15 (10)	0.304
	Caffeine use after 15:00	47 (27)	35 (24)	0.522
	Diuretics between 16:00-07:00	15 (9)	11 (8)	0.838
	Intravenous fluids between 20:00-07:00	107 (62)	78 (53)	0.112
	Vital checks between 22:00-06:00	93 (54)	16 (11)	<0.001
	Daytime naps (day before)	89 (51)	69 (47)	0.435
**Physical and mental health status,** median [IQR]				
	Depression (VAS)	3 [1-6]	3 [1-6]	0.558
	Anxiety (VAS)	1 [1-4]	2 [1-5]	0.064
	Pain (VAS)	3 [1-6]	3 [1-6]	0.138
	Modified Early Warning Score	0 [0-1]	0 [0-1]	0.130

Abbreviations: IQR, interquartile range; y, years; n, number; %, percentage; VAS, Visual Analogue Scale
All data were collected after the first night of study participation.

© 2022 van den Ende ES et al. *JAMA Network Open.*

eTable 5. Clinical outcomes					
			Control group (n=178)	Intervention group (n=150)	p-value
**After first night of study**					
	Physical and mental health status, median [IQR]				
		Depression (VAS)	3 [1-6]	3 [1-6]	0.558
		Anxiety (VAS)	1 [1-4]	2 [1-5]	0.064
		Pain (VAS)	3 [1-6]	3 [1-6]	0.238
		MEWS	0 [0-1]	0 [0-1]	0.130
**After thirty days**					
	Length of hospital stay in days, median [IQR]		4 [3.0-6.0]	4 [2.5-6.5]	0.506
	Place of care after hospital discharge				
		Home without home care	129 (72)	119 (80)	.
		Home with home care	7 (4)	0 (0)	.
		Inpatient rehabilitation facility	3 (2)	1 (1)	.
		Hospice-palliative home care	0 (0)	1 (1)	.
		Care home	2 (1)	0 (0)	.
		Skilled nursing facility	6 (3)	5 (3)	.
		Transfer to other hospital	1 (1)	0 (0)	.
		Passed away in hospital	0 (0)	2 (1)	.
		Missing	30 (17)	22 (15)	.
	Incidence of unplanned ICU admission		2 (1%)	0 (0)	0.334
	Incidence of delirium		4 (2)	5 (3)	0.674
	Hospital readmission		40 (22)	37 (25)	0.940

Abbreviations: IQR, interquartile range; ICU, Intensive Care Unit; VAS, Visual Analogue Scale; MEWS, Modified Early Warning Score

Data are presented as n (%) unless indicated otherwise

© 2022 van den Ende ES et al. *JAMA Network Open.*
